# Combining ^68^Ga-PSMA-PET/CT-Directed and Elective Radiation Therapy Improves Outcome in Oligorecurrent Prostate Cancer: A Retrospective Multicenter Study

**DOI:** 10.3389/fonc.2021.640467

**Published:** 2021-05-10

**Authors:** Simon Kirste, Stephanie G. C. Kroeze, Christoph Henkenberens, Nina-Sophie Schmidt-Hegemann, Marco M. E. Vogel, Jessica Becker, Constantinos Zamboglou, Irene Burger, Thorsten Derlin, Peter Bartenstein, Juri Ruf, Christian la Fougère, Matthias Eiber, Hans Christiansen, Stephanie E. Combs, Arndt-Christian Müller, Claus Belka, Matthias Guckenberger, Anca-Ligia Grosu

**Affiliations:** ^1^ Department of Radiation Oncology, Medical Center—University of Freiburg, Faculty of Medicine, University of Freiburg, Freiburg, Germany; ^2^ German Cancer Consortium (DKTK), Partner Site Freiburg, Freiburg, Germany; ^3^ Department of Radiation Oncology, University Hospital Zürich, University of Zurich, Zurich, Switzerland; ^4^ Department of Radiotherapy and Special Oncology, Medical School Hannover, Hannover, Germany; ^5^ Department of Radiation Oncology, University Hospital LMU Munich, Munich, Germany; ^6^ German Cancer Consortium (DKTK), Partner Site Munich, Munich, Germany; ^7^ Department of Radiation Oncology, Technical University Munich, Munich, Germany; ^8^ Institute of Radiation Medicine (IRM), Helmholtz Zentrum Munich, Oberschleissheim, Germany; ^9^ Department of Radiation Oncology, University Hospital Tübingen, Tübingen, Germany; ^10^ Department of Nuclear Medicine, University Hospital Zürich, University of Zurich, Zurich, Switzerland; ^11^ Department of Nuclear Medicine, Hannover Medical School, Hannover, Germany; ^12^ Department of Nuclear Medicine, University Hospital LMU Munich, Munich, Germany; ^13^ Department of Nuclear Medicine, Medical Center—University of Freiburg, University of Freiburg, Freiburg, Germany; ^14^ Department of Nuclear Medicine and Clinical Molecular Imaging, University Hospital Tübingen, Tübingen, Germany; ^15^ German Cancer Consortium (DKTK), Partner Site Tübingen, Tübingen, Germany; ^16^ Department of Nuclear Medicine, Technical University Munich, Munich, Germany

**Keywords:** metastasis-directed radiotherapy, oligorecurrent, prostate cancer, elective prostate bed radiotherapy, radiotherapy, elective nodal radiotherapy

## Abstract

**Background:**

In case of oligo-recurrent prostate cancer (PC) following prostatectomy, ^68^Ga-PSMA-PET/CT can be used to detect a specific site of recurrence and to initiate metastasis-directed radiation therapy (MDT). However, large heterogeneities exist concerning doses, treatment fields and radiation techniques, with some studies reporting focal radiotherapy (RT) to PSMA-PET/CT positive lesions only and other studies using elective RT strategies. We aimed to compare oncological outcomes and toxicity between PET/CT-directed RT (PDRT) and PDRT plus elective RT (eRT; i.e. prostate bed, pelvic or paraaortal nodes) in a large retrospective multicenter study.

**Methods:**

Data of 394 patients with oligo-recurrent ^68^Ga-PSMA-PET/CT-positive PC treated between 04/2013 and 01/2018 in six different academic institutions were evaluated. Primary endpoint was biochemical-recurrence-free survival (bRFS). bRFS was analyzed using Kaplan–Meier survival curves and log rank testing. Uni- and multivariate analyses were performed to determine influence of treatment parameters.

**Results:**

In 204 patients (51.8%) RT was directed only to lesions seen on ^68^Ga-PSMA-PET/CT (PDRT), 190 patients (48.2%) received PDRT plus eRT. PDRT plus eRT was associated with a significantly improved 3-year bRFS compared to PDRT alone (53 vs. 37%; p = 0.001) and remained an independent factor in multivariate analysis (p = 0.006, HR 0.29, 95% CI 0.12–0.68). This effect was more pronounced in the subgroup of patients who were treated with PDRT and elective prostate bed radiotherapy (ePBRT) with a 3-year bRFS of 61% versus 22% (p <0.001). Acute and late toxicity grade ≥3 was 0.8% and 3% after PDRT plus eRT versus no toxicity grade ≥3 after PDRT alone.

**Conclusions:**

In this large cohort of patients with oligo-recurrent prostate cancer, elective irradiation of the pelvic lymphatics and the prostatic bed significantly improved bRFS when added to ^68^Ga-PSMA-PET/CT-guided focal radiotherapy. These findings need to be evaluated in a randomized controlled trial.

## Introduction

Primary, curative treatment of localized prostate cancer (PC) can be performed with either radical prostatectomy (RP) or radiation therapy (RT). In the case of a biochemical relapse after RP, which occurs in up to 50% depending on stage and adverse factors (1, 2), salvage RT of the prostatic bed is performed to achieve long-term disease control in terms of biochemical relapse-free survival (bRFS) as well as cancer specific survival (3).

With the development of improved imaging techniques such as positron emission tomography/computed tomography (PET/CT) it is possible to perform molecular staging before salvage RT and to tailor the radiation volume to the recurrence detected by PET/CT without irradiating elective areas. Furthermore, the implementation of new tracers, such as prostate-specific membrane antigen (PSMA) has significantly improved detection rates for recurrences even at low prostate-specific antigen (PSA) values enabling new treatment concepts (4). The rationale for metastases-directed therapy (MDT) is to eradicate all visible disease locations with high doses to delay the use of androgen-deprivation-therapy (ADT) or even prolong progression-free survival while limiting side effects that could potentially occur by the use of larger radiation treatment fields (5).

Two randomized phase II trials evaluated the role of MDT versus observation in patients with oligo-recurrent PC (6, 7). In the STOMP trial the primary endpoint, median ADT-free survival, was improved from 13 to 21 months with MDT and in the ORIOLE trial MDT was associated with an improved progression-free survival (HR 0.3, 95% CI 0.11–0.81).

In spite of the growing interest in treating oligo-recurrent patients with MDT there is no consensus on the optimal target volumes, doses and techniques for RT in this setting (8). So far, guidelines from different collaborative groups on postoperative RT recommend RT of the prostate bed in case of a biochemical recurrence (9–11). Nevertheless, it remains unclear if the prostate bed or other elective areas should be irradiated in the oligo-metastatic setting.

The aim of this study was to analyze the outcome and toxicity of PET/CT-directed RT (PDRT) versus PDRT plus elective RT (eRT) in oligo-metastatic PC. Specifically, in patients without macroscopically local recurrence after RP, we evaluated the impact of PDRT alone versus elective prostate bed RT (ePBRT) plus PDRT.

## Methods

### Patient Population

Data of 394 patients from six different academic centers that were treated with curatively intended salvage RT for oligo-recurrent prostate cancer with PSMA-ligand positive lesions on ^68^Ga-PSMA-PET/CT were evaluated between April 2013 and January 2018. All patients had prior RP with no evidence of distant metastases at initial diagnosis. According to clinical practice in each institution patients were discussed in a multidisciplinary tumor board before the initiation of oligometastatic treatment. Main inclusion criteria were: biochemical recurrence with either local manifestations (prostate bed), nodal or extra nodal metastases on ^68^Ga-PSMA-PET/CT; irradiation to all PSMA-ligand positive lesions with curative intent. Any serum prostate specific antigen (PSA) level at the time of ^68^Ga-PSMA-PET/CT was accepted. In line with the concept of oligo-metastatic disease patients with a maximum of five visceral and/or bone metastases were included. Exclusion criteria were: Recurrences under active ADT, previous chemotherapy for PC or history of previous RT of the prostate bed and/or pelvic lymph nodes after an earlier biochemical recurrence following RP. This retrospective multicenter study was approved by the institutional review board of the principal investigator´s institution and by the respective review boards of collaborating institutions.

### ^68^Ga-PSMA-PET/CT and Radiation Therapy (RT)

Pre-RT staging was performed by PET imaging with ^68^Ga labeled PSMA-11 ligands in conjunction with either contrast-enhanced or low-dose computed tomography with imaging approximately 1 h after intravenous radiotracer administration according to local clinical practice and in accordance with the joint EANM and SNMMI guidelines (12). To reduce activity in the urinary system, furosemide was injected intravenously 30 min prior to the tracer injection and patients were asked to void prior to the scan. The co-registered PET and CT datasets were analysed using predefined PET window settings (e.g. inverted gray scale, SUV range: 0 to 10). A PSMA-positive lesion was visually defined as focal tracer accumulation greater than normal or physiological local background activity. All lesions were irradiated using conventionally fractionated RT or stereotactic body radiotherapy (SBRT). Dose escalation was performed by a sequential or simultaneous integrated boost technique (SIB).

**Figure 1:**
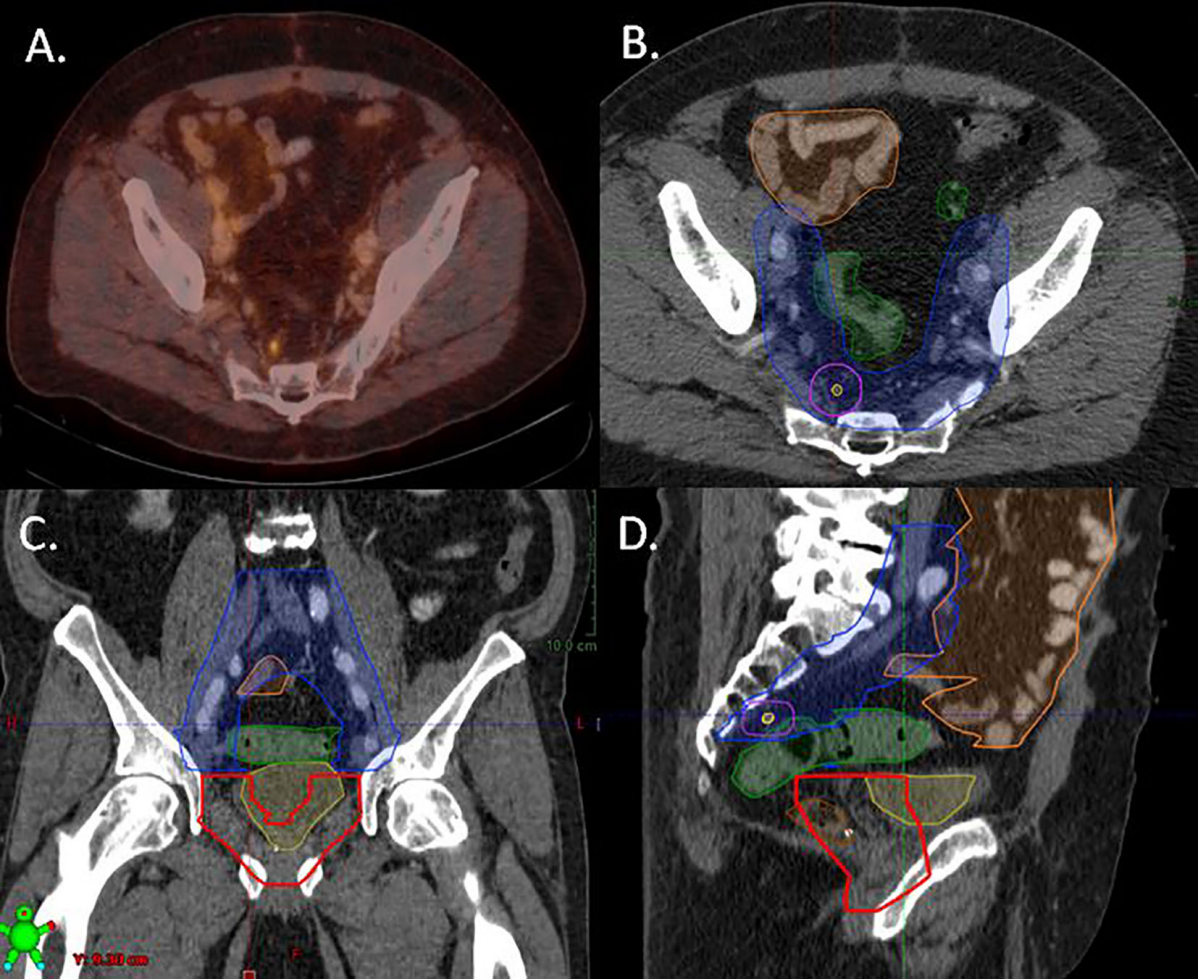
Treatment plan of a patient with a presacral lymph node recurrence on PSMA-PET/CT illustrating the different target volume concepts. The patient was treated with elective prostate bed RT (ePBRT) and elective bilateral lymphatic RT with dose escalation to the PET/CT positive lymph node (PDRT). **(A)** Fused PET/CT image, **(B)** axial plain, **(C)** coronar plain, **(D)** sagittal plain. Yellow line: PSMA-PET/CT positive lymph node; lila line: planning target volume for PSMA-PET/CT positive lymph node; Blue line: elective lymph node RT volume including presacral and bilateral internal iliac nodes; red line: elective prostate bed RT; Green line: organ at risk (sigma); Orange line: organ at risk (small bowel).

Treatment technique, target volume concept, dose per fraction, total dose, image guidance and type and length of concomitant ADT treatment were at the discretion of each institution. The prescribed RT dose was converted to EQD2 in Gy using an α/β ratio of 1.5 Gy for prostate cancer. For the purpose of this study two basic target volume concepts were defined: One group that received RT directed to PSMA-expressing lesions only (PDRT) and one group that received PDRT plus RT of elective areas (eRT). Elective areas included the prostate bed, pelvic or paraaortal lymphatics. The respective treatment fields are illustrated in **Figure 1**.

Patients without PET positive local recurrence in the prostate bed were evaluated separately: the group receiving elective prostate bed RT (ePBRT) was compared with patients not receiving ePBRT.

### Study End Points and Statistical Analysis

Biochemical recurrence-free survival (bRFS) was the primary endpoint. In accordance with the EAU and ASTRO/AUA guidelines an increase of serum PSA value of ≥0.2 ng/ml above the nadir following definitive treatment of ^68^Ga-PSMA-PET/CT recurrences was considered an event (9, 13). In case serum PSA-levels did not respond to RT, pre-RT levels with a rise of ≥0.2 ng/ml were used. Time to event was calculated from the last day of RT. BRFS was analyzed using Kaplan–Meier survival curves and log rank testing to compare differences between survival curves. Uni- and backward multivariate analyses were performed to determine influence of treatment parameters on bRFS. A p value of <0.05 was considered statistically significant. Variables included were initial T-, and N-stage, initial risk score, initial resection margins, initial PSA, initial Gleason score, PSA before start of salvage RT, PSA response, local recurrence of the prostate bed, N-, and M-stage at time of recurrence, RT of elective areas and additive ADT. Acute and late gastro-intestinal and genito-urinary toxicities were analyzed using the National Cancer Institute Common Terminology Criteria for Adverse Events (CTCAE) v4.03. Survival curves were generated by the Kaplan–Meier method using SPSS v27.0 statistic software package (IBM, USA). Follow-up after RT was done according to the institutions guidelines including regular PSA measurements.

## Results

### Patient Characteristics

The median age for the whole population at the time of 68Ga-PSMA-PET was 69 years (range, 46–95). The majority of patients (96.1%) had high risk or very high risk features according to D’Amico classification, 162 (41.1%) patients had a Gleason Score of ≥8 and 120 (30.5%) patients presented with lymph node positive disease. Median time to biochemical recurrence after RP was 15 months (range, 0–196). The median PSA value at the time of RT was 1.2 ng/ml (0.04–47.5). Additive ADT was given in 130 patients. Detailed patient characteristics can be found in **Table 1**.

**Table 1:** Patient characteristics.

	Whole cohortn = 394	PDRTn = 204	PDRT plus eRTn = 190
**Age at primary treatment** **(y) (median, range)**	66 (46–82)	65.5 (46–81)	66 (46–82)
**Initial PSA (ng/ml) (median, range)**	11 (2.1–657.20)	9.8 (3.1–657.2)	13.7 (2.8–368)
**Initial T stage**			
** pT1c**	8 (2.0)	7 (3.4)	1 (0.5)
** pT2a**	15 (3.8)	11 (5.4)	4 (2.1)
** pT2b**	11 (2.8)	7 (3.4)	4 (2.1)
** pT2c**	126 (32.0)	77 (37.7)	49 (25.8)
** pT3a**	90 (22.8)	42 (20.6)	48 (25.3)
** pT3b**	134 (34.0)	57 (27.9)	77 (40.5)
** pT4**	9 (2.3)	2 (1.0)	7 (3.7)
** Tx**	1 (0.3)	1 (0.5)	0
**Initial N stage**			
** pN0**	261 (66.2)	162 (79.4)	100 (52.6)
** pN1**	120 (30.5)	35 (17.2)	84 (44.2)
** Nx**	13 (3.3)	7 (3.4)	6 (3.2)
**Initial Gleason score**			
** 6**	21 (5.3)	19 (9.3)	2 (1.1)
** 7a**	82 (20.9)	47 (23.0)	36 (18.9)
** 7b**	127 (32.2)	67 (32.8)	60 (31.6)
** 8**	51 (12.9)	23 (11.4)	28 (14.7)
** 9**	108 (27.4)	47 (23.0)	60 (31.6)
** 10**	3 (0.8)	0	3 (1.6)
** Unknown**	2 (0.5)	1 (0.5)	1 (0.5)
**Initial risk group**			
** Intermediate**	14 (3.6)	11 (5.4)	3 (1.6)
** High risk**	379 (96.1)	192 (94.1)	187 (98.4)
** Unknown**	1 (0.3)	1 (0.5)	0
**Surgical margins**			
** R0**	217 (55.3)	127 (62.3)	90 (47.4)
** R1/R2**	166 (42.4)	66 (32.4)	100 (52.6)
** Rx**	11 (2.3)	11 (5.3)	0
**Time to biochemical recurrence (mo) (median, range)**	15 (0–196)	27 (0–196)	5 (0–166)
**PSA at time of MDT (ng/ml) (median, range)**	1.2 (0.04–47.5)	1.5 (0.05–47.5)	0.9 (0.04–40.1)

### ^68^Ga-PSMA-PET/CT Before Radiation Therapy (RT)

**Figure 2:**
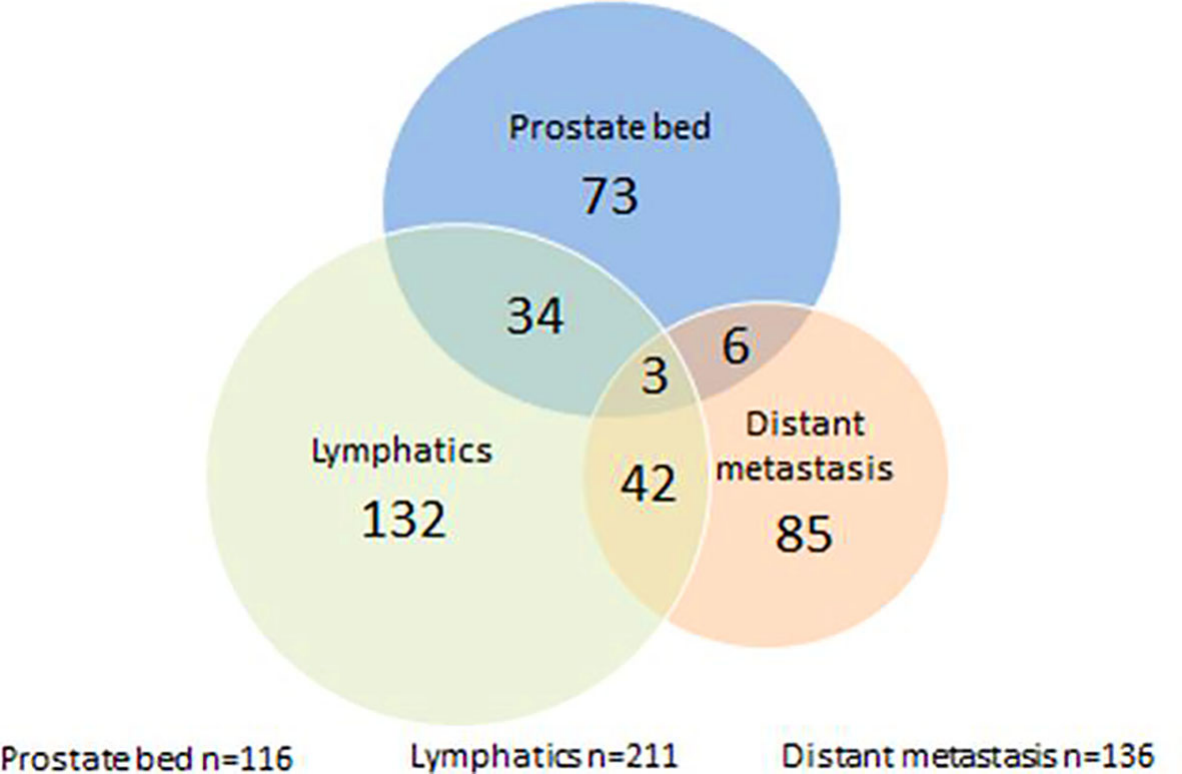
Pre-radiotherapy PSMA-PET/CT findings; n = 394.

**Figure 2** depicts the pre-RT ^68^Ga-PSMA-PET/CT findings. One hundred and sixteen of 394 patients (29.4%) had a recurrence in the prostate bed, 211 of 394 (53.6%) had a recurrence in lymph nodes and 136 of 394 patients (34.5%) had distant metastases (**Figure 2**). According to ^68^Ga-PSMA-PET/CT, recurrence was localized in 73 patients in the prostate bed only, in 34 patients in prostate bed and lymph nodes and in 132 patients in lymph nodes only. 134 patients presented with distant metastases and 42 patients with distant metastases and lymph nodes metastases.

### Radiation Therapy Target Volume and Dose

Two hundred four patients (51.8%) were treated with PDRT and 190 patients (48.2%) received PDRT plus eRT. Areas of elective RT included the prostate bed in 117 of 190 patients (61.6%), pelvic lymphatics in 163 of 190 patients (85.8%) and paraaortic lymph nodes in 21 of 190 patients (11.1%) (**Table 2**).

**Table 2:** Patterns of recurrence and elective treatment areas.

**PSMA pos. Local recurrence in prostate bed**	
** no**	278 (70.6)
** yes**	116 (29.4)
**PSMA-positive recurrences lymph nodes (n):**	
** N0**	183 (46.4)
** N1**	211 (53.6)
**PSMA-positive distant metastasis (n):**	
** M0**	258 (65.5)
** M1a**	57 (14.3)
** M1b**	72 (18.1)
** M1c**	7 (1.8)
**Elective RT volumes:**	
** no**	204 (51.8)
** yes**	190 (48.2)
** Prostate bed only**	23 (12.1)
** Prostate bed+lymphatics**	94 (49.5)
** Lymphatics**	73 (38.4)
**RT technique**	
** Conventional**	205 (52.0)
** Conventional with SIB**	130 (33.0)
** SBRT**	38 (9.6)
** Conventional with SBRT**	21 (5.4)
**Elective volume dose (EQD2/1.5 Gy) (median, range)**	
** Prostate bed**	66 (47.5-70)
** Pelvic lymphatics**	47.5 (42–56)
**Additive ADT**	
** no**	262 (66.5%)
** yes**	130 (33.0%)
** unknown**	2 (0.5%)

In patients without macroscopic recurrence in the prostate bed, elective RT of the prostate bed (ePBRT) was performed with a median dose of 66 Gy (range, 47.5–70 Gy) in single doses of 1.8–2 Gy. If pelvic lymphatics were electively irradiated the median dose was 47.5 Gy (range, 36–56/EQD 2/1.5 Gy). ^68^Ga-PSMA-PET/CT-positive local recurrences within the prostate bed were treated with a median dose of 71.2 Gy (range, 62.6–83/EQD 2/1.5 Gy), PSMA PET-positive pelvic lymph nodes with 59.4 Gy (range, 46–85/EQD 2/1.5 Gy) and paraaortic lymph nodes with 55 Gy (50–99/EQD 2/1.5 Gy).

Most patients were treated with conventionally fractionated RT 205 (52.0%) or conventionally fractionated RT with a simultaneous integrated boost (SIB) technique 130 (33.0%). SBRT was used in 38 (9.6%) and combined SBRT and conventional RT in 21 (5.4%) patients.

### Clinical Outcomes

**Figure 3:**
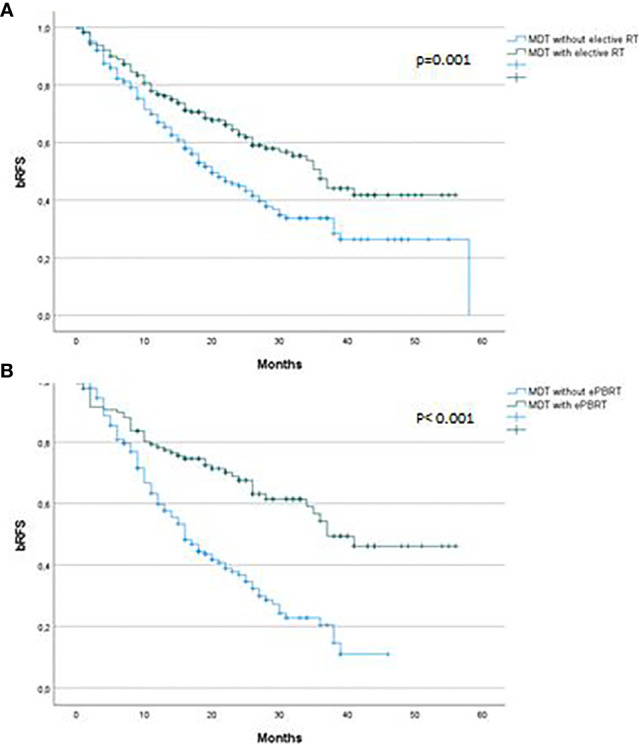
Biochemical recurrence free survival after ^68^Ga-PSMA-PET CT-directed radiotherapy of prostate cancer recurrences **(A)** stratified by elective RT versus no elective RT, **(B)** stratified by elective RT to prostate bed versus no elective RT to prostate bed. bRFS, Biochemical recurrence free survival; MDT, Metastasis-directed therapy; ePBRT, elective prostate bed radiotherapy.

The majority of patients, 364 of 394 (92.4%) showed a decrease of the PSA value 2 months after RT with a median PSA nadir of 0.07 ng/ml (range, 0.01–13.71). Median follow-up was 28 months (range, 1–71). In total, 193 of 394 patients (49.0%) had a biochemical recurrence. Median bRFS was 27 months (**Figure 3**).

Patients who were treated with PDRT had a 3-year bRFS of 37% compared to 53% in patients who received PDRT plus eRT (p = 0.001). Median bRFS was 20 vs. 36 months. Other significant factors in univariate analysis were initial T status, initial lymph node status, Gleason score, local recurrence in the prostate bed, M status at time of ^68^Ga-PSMA-PET/CT, PSA value at the start of RT, RT technique, additive ADT and area of elective RT. Initial T stage (<T2c vs. ≥T2c; p = 0.035), M status at time of recurrence, PSA value at the start of RT, additive ADT and elective RT (p = 0.005, HR 0.29, 95% CI 0.12–0.68) were independent predictors of bRFS in multivariate analysis (**Table 3**).

**Table 3:** Univariate and multivariate Cox regression analysis determining independent factors influencing biochemical recurrence-free survival for **(A)** whole cohort and **(B)** Prostate bed negative on PSMA-PET/CT.

A. Whole cohort n = 394			
Variables	Univariate	Multivariate	HR (95% CI)
	P value	P value	
Time to BR after primary therapy (≤15, >15 mo)	0.677		
Initial T-status (≤T2c, >T2c)	**0.018**	0.020	1.49 (1.07–2.08)
Initial N-status	**0.028**		
Gleason Score (≤7a, 7b, ≥8)	**0.025**		
Initial PSA (≤10ng/ml, 10-20ng/ml, >20ng/ml)	0.121		
Initial risk score	0.689		
Local recurrence prostate bed	**0.003**		
M-status at time of recurrence	**<0.001**	0.001	1.95 (1.32–2.86)
N-status at time of recurrence	0.605		
PSA at time of SRT (≤0.5 ng/ml, >0.5 ng/ml)	**0.005**	0.009	1.53 (1.11–2.10)
Resection margins (R0 vs. R1–2)	0.072		
Additive ADT	**<0.001**	<0.001	0.36 (0.24–0.53)
Elective RT vs. No elective RT	**<0.001**	0.006	0.33 (0.15–0.73)
Area of elective RT (prostate bed, lymphatics)	**<0.001**	0.006	1.76 (1.03–3.83)
Radiotherapy technique (Conventional vs. SBRT)	**<0.001**		
**B. Prostate bed negative on PSMA-PET/CT n = 278**			
**Variables**	**Univariate**	**Multivariate**	**HR (95% CI)**
	**P value**	**P value**	
Time to BR after primary therapy (≤15, >15mo)	0.701		
Initial T-status (≤T2c, >T2c)	**0.035**	0.009	1.67 (1.14–2.44)
Initial N-status	0.063		
Gleason Score (≤7a, 7b, ≥8)	0.31		
Initial PSA (≤10 ng/ml, 10–20 ng/ml, >20 ng/ml)	0.633		
Initial risk score	0.431		
M-status at time of recurrene	**<0.001**	0.002	2.01 (1.32–3.34)
N-status at time of recurrence	**0.036**		-
PSA at time of SRT (≤0.5 ng/ml, >0.5 ng/ml)	**0.001**	0.019	1.56 (1.08–2.25)
Resection margins (R0 vs. R1–2)	0.107		
Additive ADT	**<0.001**	<0.001	0.35 (0.22–0.55)
Elective RT prostate bed	**<0.001**	0.020	0.59 (0.37–0.92)
Radiotherapy technique (Conventional vs. SBRT)	**0.001**		

In a next step we aimed to analyze the influence of elective prostate bed RT (ePBRT) looking only at 278 patients without ^68^Ga-PSMA-PET/CT positive prostate bed recurrence. Of these 278 patients, 117 (42.1%) were treated with ePBRT plus PDRT. The 3-year bRFS was 22% and 61% for PDRT only and ePBRT plus PDRT, respectively (p <0.001). Median bRFS was 16 vs. 37 months. This was also significant in multivariate analysis (p = 0.02, HR 0.59, 95% CI 0.37–0.92). Other factors that were significantly associated with bRFS in univariate analysis were initial T stage, N and M stage at the time of recurrence as well as PSA at the time of sRT (≤0.5 ng/ml vs. ≥0.5 ng/ml), additive ADT and technique of RT (conventional vs. SBRT). In multivariate analysis, in addition to ePBRT and additive ADT (p <0.001), initial T (p = 0.009) and M stage (p = 0.002) were significantly correlated to bRFS. RT dose to the prostate bed or lymph nodes had no influence on bRFS (**Table 3**). We performed an additional analysis excluding M positive patients. Elective RT remained a significant factor for bRFS in this cohort (p = 0,003) with a median bRFS of 41 versus 26 months for elective and no elective RT respectively.

**Figure 4:**
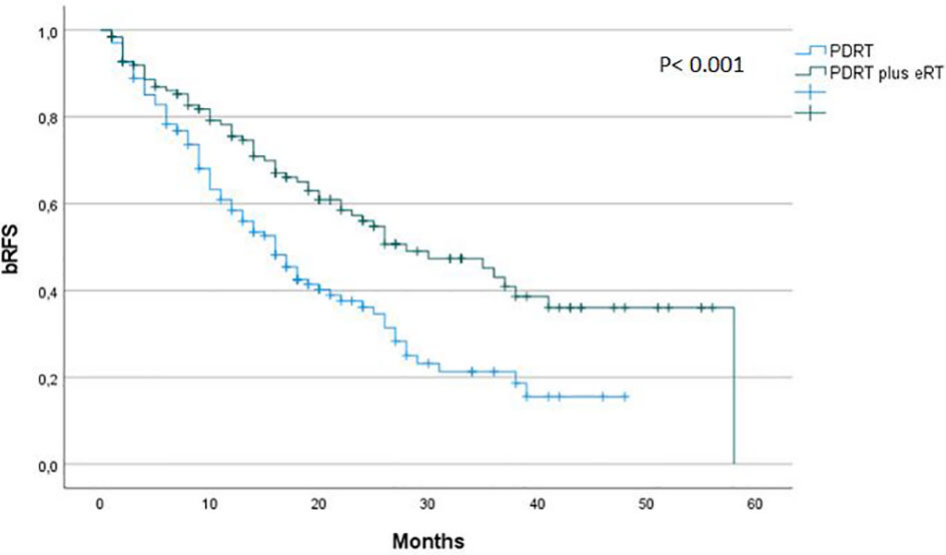
Biochemical recurrence free survival after ^68^Ga-PSMA-PET CT-directed radiotherapy of prostate cancer recurrences in patients not receiving ADT stratified by elective RT versus no elective RT. bRFS, Biochemical recurrence free survival; PDRT, PET/CT-directed radiotherapy; eRT, elective radiotherapy.

To investigate the impact of elective RT independently of ADT we performed an additional analysis excluding patients who received ADT (n = 130). Median bRFS was 16 versus 28 months for patients receiving PDRT only and ePBRT plus PDRT, respectively (p <0.001) (**Figure 4**).

### Toxicity

Overall RT was well tolerated with very few acute gastrointestinal (GI) and genitourinary (GU) toxicities. Acute grade 3 toxicity was observed in two patients (diarrhea, lymphedema). Acute grade 2 GI and GU toxicity were observed in 14 and 8.4%, respectively. Late GI toxicity (only grade 2) was observed in 3% and late GU toxicity in 10.9% of patients consisting of seven patients with grade 3 toxicity.

We analyzed if elective RT was associated with an increase in toxicity. Although toxicities were low overall there were significantly more acute (grade 2: 8.8% vs. 31.5%, grade 3: 0% versus 0.4%) and late (grade 2: 1.9% vs. 19.2%, grade 3: 0% versus 3%) GI and GU side effects in patients receiving elective RT (p = 0.001) (**Table 4**).

**Table 4:** Acute and late GI and GU toxicity (≥grade 2) by treatment volume concept according to CTCAE v4.03.

Toxicity	Acute toxicity			
	PDRT n (%)		PDRT + eRT n (%)	
	**Grade 2**	**Grade 3**	**Grade 2**	**Grade 3**
**GU**	2 (1.3)	0	31 (13.2)	0
**GI**	12 (7.5)	0	43 (18.3)	1 (0.4)
**Other**	0	0	0	1 (0.4)
	**Late toxicity**			
	**PDRT n (%)**		**PDRT + eRT n (%)**	
	**Grade 2**	**Grade 3**	**Grade 2**	**Grade 3**
**GU**	1 (0.6)	0	35 (14.9)	7 (3.0)
**GI**	2 (1.3)	0	10 (4.3)	0
**Other**	0	0	0	0

PDRT, PET/CT-directed radiotherapy; eRT, elective radiotherapy.

## Discussion

MDT is increasingly investigated as a treatment strategy for oligo-recurrent PC. Still the optimal treatment volume for MDT remains unclear. Some centers implement MDT using a strict definition focally treating lesions detected by PSMA-PET/CT only while other centers are using larger treatment volumes including elective areas (14). Both strategies are not included in current treatment guidelines although a substantial number of international institutions treat patients with MDT as evidenced by a consensus conference of 72 experts in 2019 (15, 16).

To our knowledge, the presented data is the largest study comparing PDRT with PDRT plus elective RT in oligo-recurrent prostate cancer using ^68^Ga-PSMA-PET/CT as the imaging modality of choice at recurrence. Our study is the first study that looks specifically at elective RT of the prostate bed. Patients receiving PDRT only progressed significantly more often and had a lower 3-year bRFS (22%) than patients receiving PDRT and elective prostate bed RT (ePBRT) (3-year bRFS 61%; p <0.001). This effect proved to be significant in multivariate analysis as well.

A possible explanation for this finding is the limited sensitivity of imaging in the detection of microscopic disease. Though molecular imaging with Choline or PSMA PET/CT has substantially improved detection rates up to 76% for PSA values <1 ng/ml (17), we probably still underestimate the true extent of disease. In a very recent study by Fossati et al. the number of positive lymph nodes found on histology exceeded the number of PET/CT positive lymph nodes (18). This effect was less pronounced for PSMA—than for Choline—PET. The moderate sensitivity of PSMA-PET/CT for the detection of pelvic lymph node metastasis was also shown in series of patients who underwent PET/CT before extended lymph node dissection. The sensitivity ranges from 33–100% and per-node sensitivity is in the range of 24–66% (19). Another demonstration for the underestimation of nodal disease in PET/CT is a study by Rischke et al. (20). In this study patients were treated with additional RT after PET/CT guided salvage lymph node resection. By the addition of RT to the regions with PSMA-expressing lesions on PET/CT, 5-years-PFS was significantly improved from 26.3 to 70.7% indicating remaining micrometastasis after surgery. In analogy to nodal disease, underestimation of subclinical, microscopic disease presumably also occurs in the prostate bed being the location with the highest risk of microscopic disease after radical prostatectomy. In addition to the limited spatial resolution of PET/CT, tracer excretion *via* the bladder with subsequent blurring of the area of the prostatic fossa contributes to the difficult detection of a local recurrence in the prostate fossa.

The majority of data for elective RT comes from small retrospective series (21, 22). PFS rates at 3 years range between 49 and 75% (23). In one study by Tran et al. a 5-year bRFS rate of 43% after elective nodal RT was reported (24). The only prospective trial is the oligo-pelvis–GETUG P07 trial (24). Early toxicity results have been published last year showing low grade 3 toxicity rates even though half of the patients had a re-irradiation of the pelvis (25). Outcome data are not available yet. Comparative data for focal strategies versus elective RT are limited and of retrospective nature. In one study Lepinoy et al. evaluated outcome and toxicity in 62 nodal oligo-recurrent PC patients treated with elective nodal RT (ENRT) or involved node SBRT (26). PFS rate was significantly improved by ENRT (88.3% versus 55.3% at 3-year) while toxicities were similar. The trial that resembles our analysis the most was a large retrospective multicentre analysis by De Bleser and colleagues including 506 pelvic node oligo-recurrent PC patients (27). The primary endpoint was metastasis-free-survival (MFS) after ENRT or SBRT. ENRT was able to improve MFS for patients with a single node while MFS was similar for patients with two to five nodes. Late toxicities were higher in patients who received ENRT (16% vs. 5%). In contrast to our study RT treatment planning was based on Choline-PET/CT in the majority of patients (85%) and prostate bed irradiation was performed in only 60 of 506 patients. Additionally patients with distant metastasis were excluded.

In the current study ADT significantly improved bRFS with a 3-year bRFS rate of 62% versus 34% for patients receiving concurrent ADT to PDRT. This is in accordance with the study by Kroeze et al. with a 2-year PFS rate of 78% versus 53% (28). The additive effect of ADT was seen in patients receiving eRT or not. In patients not receiving eRT median bRFS was 16 versus 30 months for ADT versus no ADT whereas median bRFS in patients who received eRT and no ADT was 26 months. Median bRFS was not reached in the group of patients with eRT and ADT. The role of concurrent ADT in the setting of MDT still needs to be clarified. Potential improvement of survival outcomes must be weighed against increased morbidity and worse quality of life (29). There are two randomized trials showing a benefit for the addition of ADT to RT in the postoperative setting (30, 31). One trial was in the adjuvant setting (RTOG-9601) and the other trial in the salvage setting (GETUG-AFU 16). However, their results are not easily comparable as differently defined patient cohorts were included and both trials did not use pre-RT modern imaging techniques for staging making the results not comparable to the oligo-metastatic state diagnosed by PSMA-PET/CT. So far, results in the oligo-metastatic state are rare and heterogeneous. Most findings come from retrospective, small studies using Choline-PET/CT as imaging modality and varying use and duration of ADT use. The influence of systemic treatment and local treatment remains unclear in this setting. On the other hand an important aim of MDT is to postpone ADT. This was shown by Ost et al. (6). In their study MDT could prolong ADT-free survival by 8 months compared to surveillance alone. In our study the hormone-naive subgroup of patients benefited by adding elective RT areas to PDRT. Median bRFS was 16 versus 28 months in favour of PDRT plus eRT. Further prospective studies assessing the additional benefit of ADT and MDT with or without eRT are required.

Another important parameter for treatment strategy decisions is toxicity. As expected increasing the size of treatment volumes will evidently increase toxicity as shown in a study by Aiter et al. comparing prostate only versus WPRT (32) as well as in a number of other studies (9, 21). PDRT as well as PDRT plus eRT were very well tolerated in our study. Toxicities were mostly mild although PDRT plus eRT was associated with more grade 2 toxicities (8.8% compared to 31.5% and 1.9% vs. 19.2% for acute and late toxicities, respectively) and there were two acute (n = 2; diarrhea and lymphedema) and seven late (n = 7; urinary retention, cystitis) grade 3 events in the PDRT plus eRT group. Toxicity rates are comparable to the results published in the Oligo-pelvis–GETUG P07 trial and the trial by De Bleser et al. showing higher rates of GI and GU toxicity for eRT compared to focal treatment (16% vs. 5%) (33). In summary, toxicity might be slightly higher with larger treatment fields used for eRT but grade 3 toxicity rates were still low and acceptable.

The study has the known limitations inherent to a retrospective analysis, but allows the examination of real-life data in a large cohort of patients. Limitations include the following: the choice for a treatment volume concept, as well as for ADT and follow-up were not standardized and at the discretion of the treating physician implying possible bias. Also, the field for eRT was not standardized leading to potentially different treatment volumes. Further knowledge concerning the extent of the treatment field can be expected by an ongoing prospective multicenter randomized phase II trial treating patients with either MDT and ADT or MDT plus whole pelvis RT and ADT (PEACE V-STORM trial) (34). Results are eagerly awaited and can potentially help to redefine treatment guidelines for salvage RT.

## Conclusion

^68^Ga-PSMA-PET-directed RT plus eRT improves bRFS in oligo-recurrent PC patients while slightly increasing side effects. Elective prostate bed irradiation plus PDRT was associated with better bRFS compared to ^68^Ga-PSMA-PET-directed RT alone. These findings need to be confirmed in a prospective trial.

## Data Availability Statement

The raw data supporting the conclusions of this article will be made available by the authors, without undue reservation.

## Ethics Statement

The studies involving human participants were reviewed and approved by Kantonale Ethikkomission Zürich (BASEC-Nr. 2017-01499). The patients/participants provided their written informed consent to participate in this study.

## Author Contributions

SK, SGK, CH, NS-H, MV, JB, CZ, IB, TD, PB, JR, CL, ME, HC, SC, AM, CB, MG, and AG contributed to the design and implementation of the research. SK, SGK, CH, NS-H, MV, JB, CZ, PB, CL, ME, HC, SC, AM, CB, MG, and AG contributed to data collection and performed the analysis. SK, SGK, CZ, NS, MV, TD, JR, MG, and AG contributed to the writing of the manuscript. All authors contributed to the article and approved the submitted version.

## Conflict of Interest

The authors declare that the research was conducted in the absence of any commercial or financial relationships that could be construed as a potential conflict of interest.
